# Induction of IL‐1β and antimicrobial peptides as a potential mechanism for topical dithranol

**DOI:** 10.1111/exd.14310

**Published:** 2021-02-25

**Authors:** Theresa Benezeder, Ahmed Gehad, VijayKumar Patra, Rachael Clark, Peter Wolf

**Affiliations:** ^1^ Department of Dermatology Medical University of Graz Graz Styria Austria; ^2^ Department of Dermatology Brigham and Women’s Hospital Harvard Medical School Boston MA USA; ^3^ Centre International de Recherche en Infectiologie Institut National de la Santé et de la Recherche Médicale, U1111 Lyon France

**Keywords:** alopecia areata, anthralin, cytokines, psoriasis, S100a8/a9

## Abstract

Topical dithranol is effective in autoimmune conditions like alopecia areata, inducing hair regrowth in a high percentage of cases. Exact mechanisms of dithranol in alopecia areata, with seemingly healthy epidermis besides altered hair follicles, are not well understood. To better understand dithranol's mechanisms on healthy skin, we analysed its effect on normal murine as well as xenografted human skin. We found a strong increase in mRNA expression of anti‐microbial peptides (AMPs) (eg *Lcn2*, *Defb1*, *Defb3*, *S100a8*, *S100a9*), keratinocyte differentiation markers (eg *Serpinb3a*, *Flg*, *Krt16*, *Lce3e*) and inflammatory cytokines (eg *Il1b* and *Il17*) in healthy murine skin. This effect was paralleled by inflammation and disturbed skin barrier, as well as an injury response resulting in epidermal hyperproliferation, as observed in murine and xenografted adult human skin. This contact response and disturbed barrier induced by dithranol might lead via a vicious loop between AMPs such as S100a8/a9 (that led to skin swelling itself after topical application) and cytokines such as IL‐1β to an immune suppressive environment in the skin. A better understanding of the skin's physiologic response to dithranol may open up new avenues for the establishment of novel therapeutics (including AMP‐related/interfering molecules) for certain skin conditions, such as alopecia areata.

## 1 BACKGROUND

Dithranol (anthralin), introduced more than 100 years ago, has remained among the most effective topical treatment options for chronic plaque psoriasis, despite its disadvantages like irritation of perilesional skin and brownish discoloration.^1^ Moreover, topical dithranol has been investigated in the treatment of autoimmune conditions like vitiligo^2^ and alopecia areata,^3^ and found to be highly effective in the latter disease with hair regrowth in a substantial percentage of cases,^3^ matching novel therapeutics such as JAK inhibitors.^4^ Similar to topical treatment with dinitrochlorobenzene, diphenylcyclopropenone or squaric acid dibutylester for alopecia areata^5^, ^6^ a contact reaction to dithranol is followed by hair regrowth within weeks after treatment. We recently showed that dithranol exerts its anti‐psoriatic effects by directly targeting keratinocytes and their crosstalk with neutrophils and disrupts the IL‐36 inflammatory loop.^7^ We observed that in psoriasis, dithranol's therapeutic activity was completely independent of its pro‐inflammatory effect mainly on perilesional skin. However, in alopecia areata, with seemingly normal epidermis, besides altered hair follicles, dithranol's mechanisms are still not well understood.

## 2 QUESTIONS ADDRESSED

To better understand dithranol's mechanisms on healthy skin, we analysed its effect on normal murine as well as xenografted human skin. Gene expression of keratinocyte differentiation markers, anti‐microbial peptides (AMPs) and inflammatory markers was analysed in dithranol‐treated murine skin and ears. In addition, we investigated the role of AMPs in induction of inflammation on healthy skin by analysing effects of topical application of S100a8/a9 proteins on murine skin. To investigate a potential role of the aryl‐hydrocarbon receptor (AhR) in dithranol's mechanisms, we treated AhR‐deficient mice with dithranol and monitored inflammatory response.

## 3 EXPERIMENTAL DESIGN

**FIGURE 1:**
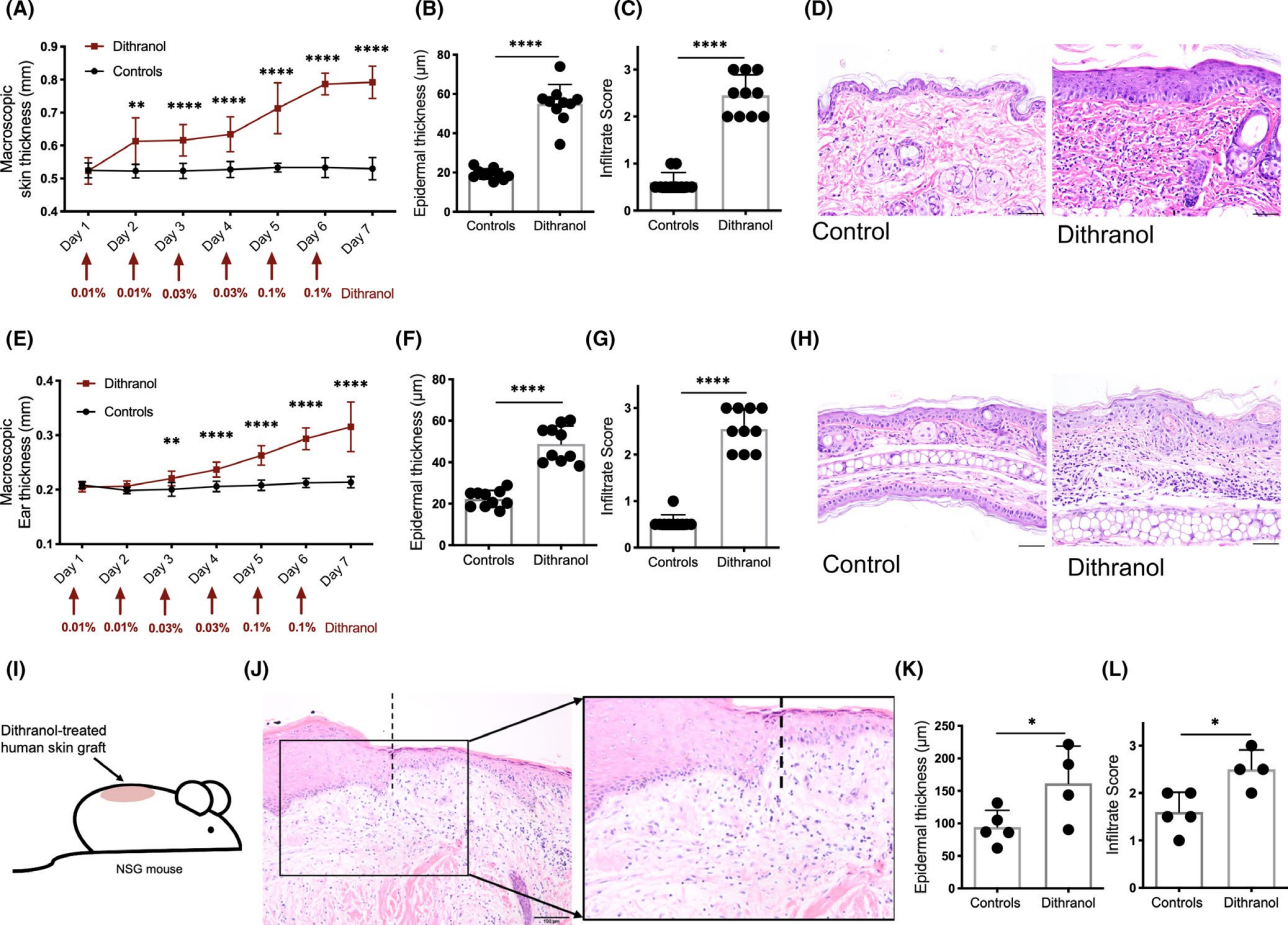
Effects of dithranol on healthy skin. A, E, Dithranol strongly increased macroscopic skin thickness of dorsal skin (A) and ears (E) in healthy mice. Arrows indicate concentration of dithranol. B‐D and F‐H, A strong increase in epidermal thickness (B, F) and cellular infiltrate score (C, G) of dithranol‐treated dorsal skin and ear skin was observed. Representative H&E images are depicted (D, H). I, Immunocompromised mice were grafted with human skin and dithranol was applied to skin grafts. J‐L, Representative H&E image showing border between human and murine skin, increased epidermal thickness (J, K) and increased cellular infiltrate (J, L) after dithranol treatment. Multiple t test and unpaired t test was used for statistics. Bars represent mean ± SD (*n* = 10 (A–H), *n* = 5 (I‐L)). *p ≤ 0.05; **p ≤ 0.01; ***p ≤ 0.001; ****p ≤ 0.0001; scale bar = 50 µm (D, H), 100 µm (J)

Dithranol was applied topically on shaved dorsal skin and ears of BALB/c mice and concentrations were increased every other day (0.01% on day 1–2, 0.03% on day 3–4 and 0.1% on day 5–6) (Figure 1). To study dithranol's effect on normal human skin, we grafted immunodeficient NSG mice with adult human skin (obtained from surgical procedures) and started dithranol application (0.1% on day 1–3, 0.3% on day 4–6) 3 weeks after engraftment (for further details, see Methods in Supporting Information). Histological analysis (epidermis thickness measurement and infiltrate score) was done 24 h after the last topical application of dithranol. For murine skin, transepidermal water loss (TEWL) and erythema index were measured 24 h after the last dithranol treatment. Based on our microarray results from dithranol‐treated psoriasis patients,^7^ we selected a panel of keratinocyte differentiation markers, anti‐microbial peptides (AMPs) and inflammatory markers (see Methods in Supporting Information) and analysed their gene expression in dithranol‐treated murine skin and ears by quantitative real‐time PCR. To investigate the role of AMPs in induction of inflammation on healthy skin, S100a8/a9 proteins were topically applied on shaved dorsal skin of C57BL/6 J mice and skin swelling as well as histological analysis was assessed after 24 h. To assess whether dithranol might act through modulation of AhR, C57BL/6 J control mice and AhR‐deficient mice were topically treated with dithranol in increasing concentrations (0.03% on day 1–2, 0.1% on day 3–4 and 0.3% on day 5–6) on shaved dorsal skin and ears and skin swelling response was monitored and histologic examinations were performed (see Methods in Supporting Information).

## 4 RESULTS

**FIGURE 2:**
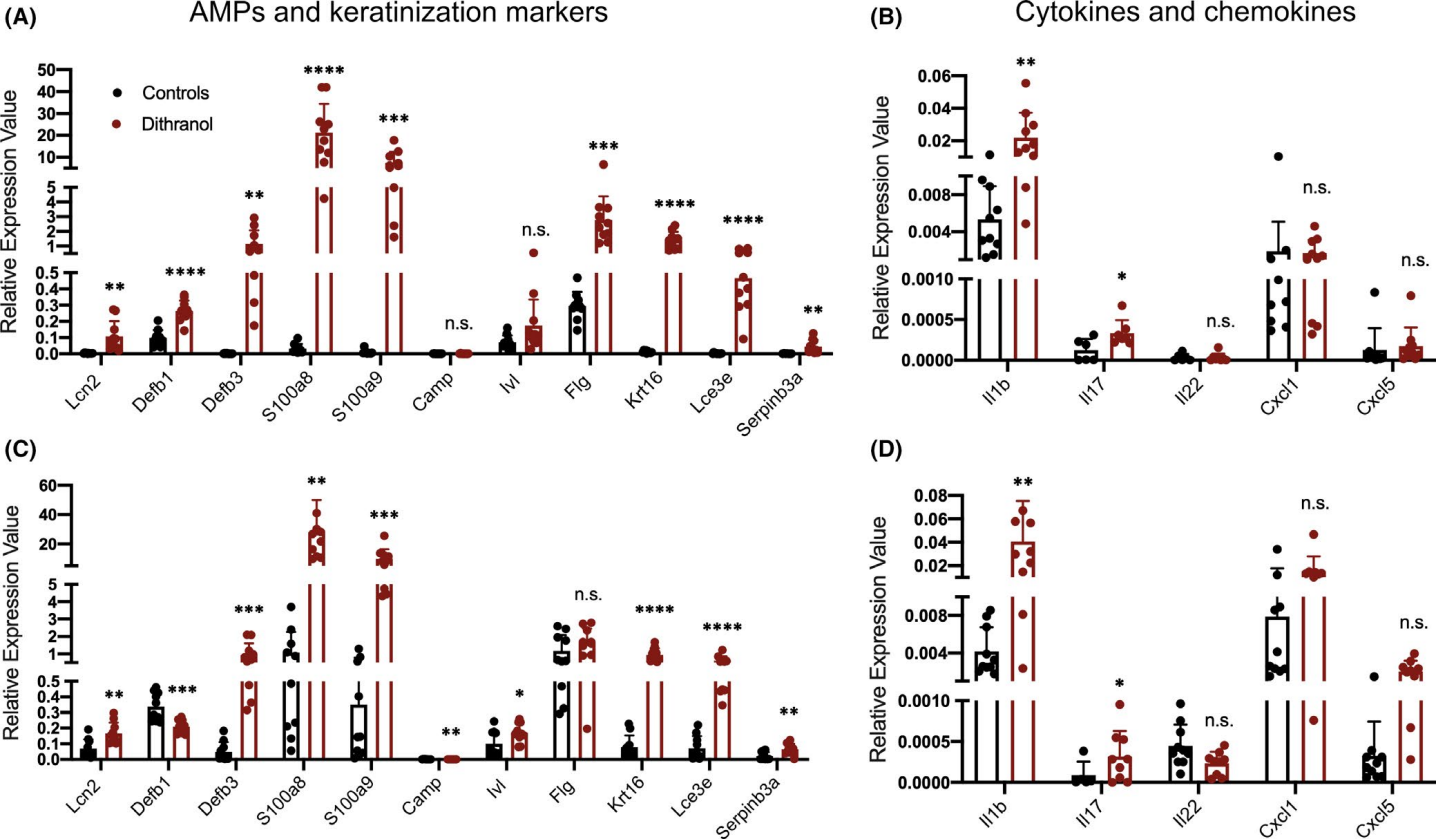
Gene expression analysis by RT‐PCR of anti‐microbial peptides (AMPs; *Lcn2*, *Defb1*, *Defb3*, *S100a8*, *S100a9*, *Camp*), keratinization markers (*Ivl*, *Flg*, *Krt16*, *Lce3e*, *Serpinb3a*) and cytokines (*Il1b*, *Il17*, *Il22*) and chemokines (*Cxcl1*, *Cxcl5*) of dithranol‐ and vehicle‐treated murine dorsal skin (A, B) and ears (C, D). Bars represent mean ± SD (*n* = 10) of relative expression values (ΔCT) normalized to *Ubc*. Unpaired *t* test was used for statistics. *p ≤ 0.05; **p ≤ 0.01; ***p ≤ 0.001; ****p ≤ 0.0001. Lcn2, lipocalin 2; Defb1, beta‐defensin 1; Defb3, beta‐defensin 3, Camp, cathelicidin antimicrobial peptide, Ivl, involucrin; Flg, filaggrin; Krt16, keratin 16; Lce3e, late cornified envelope 3e; Serpinb3a, serine peptidase inhibitor, clade B, member 3a; Il1b, interleukin 1 beta; Il17, interleukin 17; Il22, interleukin 22; Cxcl1, C‐X‐C motif ligand 1; Cxcl5, C‐X‐C motif ligand 5

**FIGURE 3:**
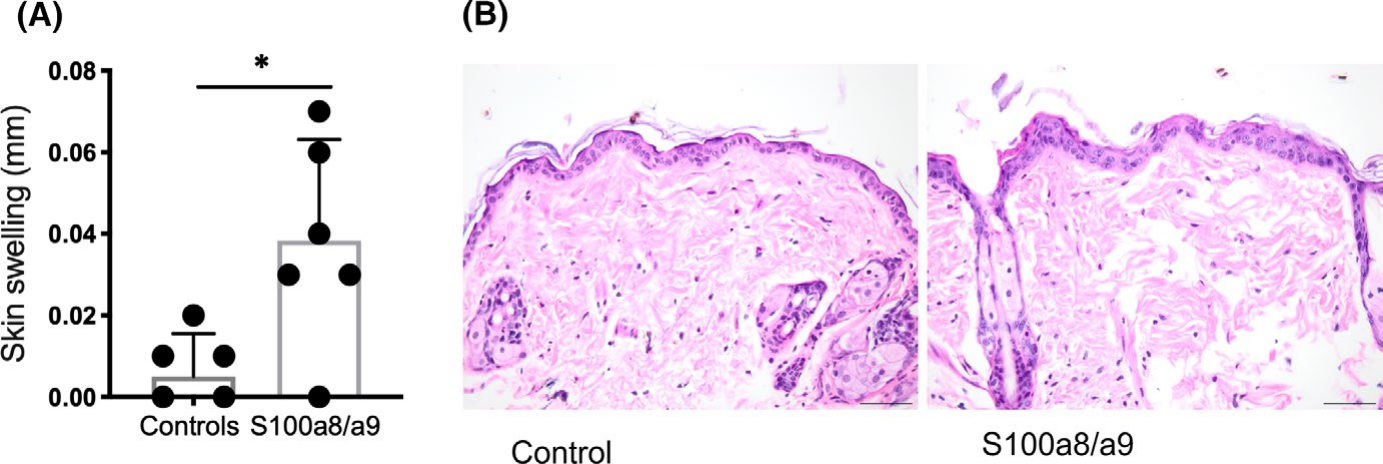
Effects of anti‐microbial peptides S100a8/a9 on healthy skin. A, Dorsal skin swelling was significantly increased 24 h after topical application of S100a8/a9 proteins. B, A slight increase in epidermal thickness and oedema in the dermis were observed. Representative H&E images are depicted. Unpaired t test was used for statistics. Bars represent mean ± SD (*n* = 5 (A)). *p ≤ 0.05; **p ≤ 0.01; ***p ≤ 0.001; ****p ≤ 0.0001; scale bar = 50 µm

Real‐time qPCR analysis of dithranol‐treated murine skin and ears revealed a strong increase in expression of AMPs (*Lcn2*, *Defb1*, *Defb3*, *S100a8*, *S100a9*), keratinocyte differentiation markers (*Serpinb3a*, *Flg*, *Krt16*, *Lce3e*) and inflammatory cytokines (*Il1b* and *Il17*) in both healthy murine dorsal (Figure 2A‐B) and ear skin (Figure 2C‐D) after 6 days of dithranol treatment. Similar effects were seen in xenotransplanted human skin, as evidenced by 5‐fold upregulation of AMPs such as *S100A12* (data not shown). The effect of dithranol on the expression of keratinocyte differentiation regulators, cytokines and AMPs was paralleled by inflammation and disturbed skin barrier, as well as an injury response resulting in epidermal hyperproliferation of the skin. We observed a significant increase in macroscopic thickness of dorsal and ear skin after a single application of dithranol and thickness steadily increased with every following application (Figure 1A, E). At day 7, transepidermal water loss (TEWL) was strongly increased and marked erythema was present (as measured by erythema index; Figure S1). In murine dorsal skin and ears, as well as in xenografted adult human skin, histologic analysis showed pronounced hyperproliferation of keratinocytes (as measured by significant increase in epidermal thickness) and noticeable infiltration of immune cells (Figure 1). Intriguingly, after dithranol application *S100a8* mRNA was most highly upregulated in murine skin (and *S100A12* in xenotransplanted human skin) and previous work indicated that the former AMP can serve as a very sensitive biomarker to detect an inflammatory response even at subclinical level and independent from the pathomechanism.^8^ Our work now indicates that S100a8/a9 may itself induce an innate (immune) response of the skin, evidenced by slight skin swelling (and slight if any increase in cellular infiltration) after its topical application to dorsal murine skin (Figure 3A). Consistently, histological analysis showed that there was a slight increase in epidermal thickness and visible oedema after application of S100a8/a9 (Figure 3B).

Our work with AhR‐deficient mice and appropriate AhR‐bearing controls revealed no differences in the response of both groups as evident by a steady increase in macroscopic skin and ear thickness (Figure S2a, c). Histological analysis showed increased epidermal thickness and intense cellular infiltrate in both AhR‐deficient mice and controls (Figure S2b, d). Thus, we conclude that dithranol does not act via modulation of AhR, at least with regard to the inflammatory response of healthy skin.

## 5 DISCUSSION

Based on these results, we hypothesize that the contact response and disturbed barrier induced by dithranol leads via a vicious loop between AMPs and cytokines such as IL‐1β to an immune suppressive environment in the skin, possibly being beneficial in alopecia areata. Keratinocytes are known to upregulate the production of cytokines and chemokines such as CCL20 but also AMPs like S100 proteins in response to injury.^9^ AMPs are not only known to eliminate pathogenic microbes but also to play a role in modulating and linking innate and adaptive immune responses^9^, ^10^ and the immunosuppressive role of IL‐1β has been well established.^11^ Intriguingly, UVB phototherapy is known for its immunosuppressive action^12^ and is not only highly effective in psoriasis but can also stimulate terminal hair regrowth in alopecia areata.^13^ Notably, UV irradiation induces the expression of various AMPs such as β‐defensins^14^ and also triggers the production of IL‐1β in keratinocytes^15^ and sebocytes,^16^ representing cells that reside in structures (ie sebaceous glands) in close proximity to hair follicles. Importantly, our results are in agreement with previously published observations, which show that dithranol has an effect on ß‐defensin expression in human keratinocytes in vitro,^17^ and increases the number of T cells and protein expression of the keratinocyte differentiation marker involucrin (as determined by immunohistochemistry) in healthy human skin.^18^ While the mechanisms of action in human skin are not known, successful treatment of alopecia areata with dithranol in animal models has been linked to reduced gene expression of TNF‐α and IFN‐γ and upregulation of IL‐1β.^19^ Keratinocytes respond to cytokines like TNF‐α, IFN‐γ and IL‐1β with increased expression of AMPs. Moreover, production of AMPs such as β‐defensin‐2 by keratinocytes is known to be induced by IL‐1β.^20^ Vice versa, AMPs such as S100a8/a9 may be involved in the upregulation of cytokines like IL‐1β. Indeed, previous work has indicated that AMPs can lead to endothelial damage by interacting with RAGE and TLR,^21^ that may provide the basis for skin oedema observed in this study. The upregulation of S100 proteins and IL‐1β in healthy skin might be responsible for dithranol's side effect (unrelated to the therapeutic effect of the treatment^7^), whereas in psoriatic lesional skin, a critical role has been assigned to the latter cytokine, being rather pro‐psoriatic.^22^ Indeed, the beneficial effect of dithranol in alopecia areata may be related to modulation of the local microbiota, consistent with the observation that certain bacterial species (such as Corynebacterium) lead to increased IL‐1β and γδT cell expansion in the skin.^22^ Various immune modulatory approaches that are in daily use for treating psoriasis are also under investigation for the treatment of alopecia areata.^23^, ^24^ What our work does not answer is whether the observed outcomes are direct or indirect effects of dithranol in causing an inflammatory response, as the set‐up of our experiments did not allow a differentiation. A better understanding of the skin's physiologic response to the traditional agent dithranol opens up new avenues for the establishment of novel therapeutics. New drugs could act through modulation of the skin microbiota and/or stimulation of the innate immune response via AMPs such as S100a8/a9 and certain cytokines such as IL‐1β, thereby being potentially beneficial in conditions such as alopecia areata and other cutaneous diseases.

Although it has been shown that IL‐1β+ cells cluster around hair follicles^25^ in AA^26^ and IL‐1β inhibits hair growth in vitro,^27^, ^28^ IL‐1β may have a beneficial effect in vivo as it is observed that dithranol,^19^ contact sensitizers and UV irradiation are effective in the treatment of AA^13^, ^29^ and induce IL‐1β expression in the skin.^15^, ^30^ Notably, intradermal injection of IL‐1β has been shown to mimic the effects of UV irradiation and allergen response by inducing emigration of LCs.^31^, ^32^, ^33^ After all, the potential role of IL‐1β in AA is also highlighted by controversial data of polymorphisms in the IL‐1β gene associated with higher susceptibility of AA.^34^, ^35^, ^36^

## CONFLICT OF INTEREST

The authors have declared that no conflict of interest exists.

## AUTHOR CONTRIBUTIONS

TB designed, planned and carried out laboratory experiments and investigations, performed statistical and data analysis, drafted figures and wrote the manuscript. PW co‐designed, planned and supervised laboratory experiments and investigations, revised the manuscript and provided valuable advice. VKP helped in performing BALB/c mouse experiments and performed C57BL/6 mouse experiment. AG performed human skin graft experiments. RC provided support in animal experimentation and valuable scientific advice. All authors read and approved the final version of the manuscript.

## Supporting information


**Figure S1.** Effect of dithranol treatment in healthy skin. Dithranol strongly increased transepidermal water loss (TEWL) (a) and erythema index (b) in dorsal skin of healthy BALB/c mice compared to vehicle‐treated controls. Unpaired t test was used for statistics. Bars represent mean±SD (n=10); *p≤0.05; **p≤0.01; ***p≤0.001; ****p≤0.0001.

**Figure S2.** Effect of dithranol on AhR‐deficient mice and AhR bearing C57BL/6J controls. a) and c) Dithranol strongly increased macroscopic skin thickness of dorsal skin (a) and ears (c) in AhR‐deficient mice and control mice. No significant difference was observed between the groups. Arrows indicate concentration of dithranol. b) and d) Increased epidermal thickness and cellular infiltrate of dithranol‐treated dorsal skin and ear skin was observed in all mice. Representative H&E images are depicted (b,d), scale bar =50µm. Multiple t test was used for statistics (n=7). AhR, aryl‐hydrocarbon receptor; n.s., not significant.

**Table S1.** RT‐PCR Primer sequences and corresponding annealing temperatures.
