# Supplementary figures and images for: Induction of IL‐1β and antimicrobial peptides as a potential mechanism for topical dithranol
Source: Exp Dermatol. 2021 Feb 25;30(6):841–6. doi: 10.1111/exd.14310 (PMC8247942; doi:10.1111/exd.14310)

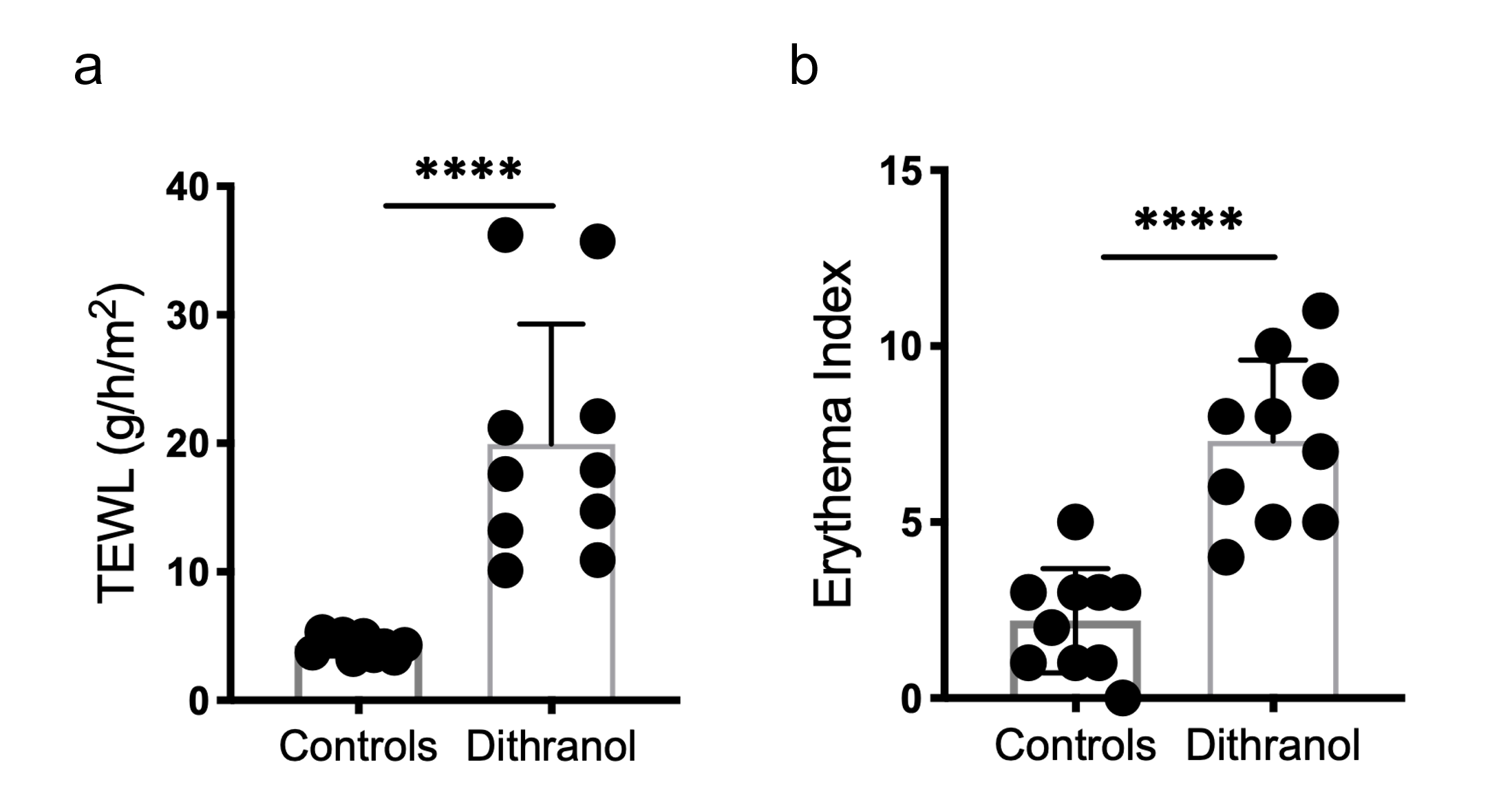

Supplement: Supplementary file 1 — Figure S1. Effect of dithranol treatment in healthy skin. Dithranol strongly increased transepidermal water loss (TEWL) (a) and erythema index (b) in dorsal skin of healthy BALB/c mice compared to vehicle‐treated controls. Unpaired t test was used for statistics. Bars represent mean±SD (n=10); *p≤0.05; **p≤0.01; ***p≤0.001; ****p≤0.0001. [file EXD-30-841-s003.png]

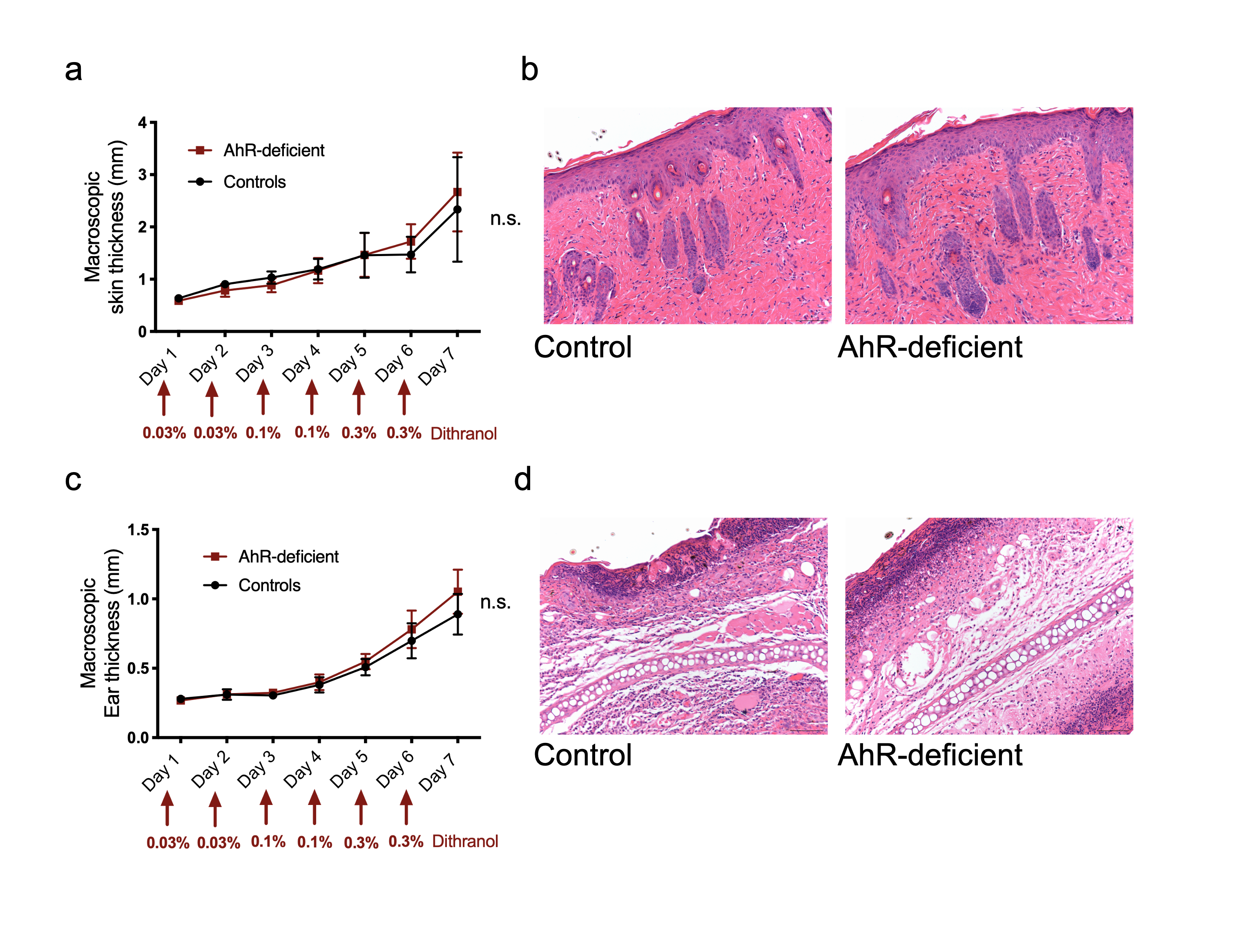

Supplement: Supplementary file 2 — Figure S2. Effect of dithranol on AhR‐deficient mice and AhR bearing C57BL/6J controls. a) and c) Dithranol strongly increased macroscopic skin thickness of dorsal skin (a) and ears (c) in AhR‐deficient mice and control mice. No significant difference was observed between the groups. Arrows indicate concentration of dithranol. b) and d) Increased epidermal thickness and cellular infiltrate of dithranol‐treated dorsal skin and ear skin was observed in all mice. Representative H&E images are depicted (b,d), scale bar =50µm. Multiple t test was used for statistics (n=7). AhR, aryl‐hydrocarbon receptor; n.s., not significant. [file EXD-30-841-s001.png]
