# Supplementary material for: Induction of IL‐1β and antimicrobial peptides as a potential mechanism for topical dithranol
Source: Exp Dermatol. 2021 Feb 25;30(6):841–6. doi: 10.1111/exd.14310 (PMC8247942; doi:10.1111/exd.14310)
Supplement: Supplementary file 3 — Table S1. RT‐PCR Primer sequences and corresponding annealing temperatures. [file EXD-30-841-s002.docx]

# **SUPPLEMENTAL INFORMATION**

**Supplemental Table 1: RT-PCR Primer sequences** and corresponding annealing temperatures.

|  |  |  |
| --- | --- | --- |
| **Primer ID** | **Sequence** | **Annealing Temperature (°C)** |
| Cxcl1 | Fw: ATCCAGAGCTTGAAGGTGTTG; | 60 |
|  | Rev: GTCTGTCTTCTTTCTCCGTTACTT |  |
| Cxcl5 | Fw: CTCAGTCATAGCCGCAACCGAGC; | 60 |
|  | Rev: CGCTTCTTTCCACTGCGAGTGC |  |
| Defb1 (Defensin beta 1) | Fw: CCAGCTGCCCATCTAATACC; | 60 |
|  | Rev: AATCCATCGCTCGTCCTTTA |  |
| Defb3 (Defensin beta 3) | Fw: TCTGACGAGTGTTGCCAATG; | 60 |
|  | Rev: ACAATCCAGTAAGTTGTTTGAGGA |  |
| Flg | Fw: GAAGGAACTTCTGGAAGGACAAC; | 60 |
|  | Rev: TCCATCAGTTCCACCATGCCTC |  |
| Il17 | Fw: GGACTCTCCA CCGCAATGA; | 60 |
|  | Rev: TCAGGCTCCCTCTTCAGGAC |  |
| Il1b | Fw: GAGTGTGGATCCCAAGCAAT; | 58 |
|  | Rev: TACCAGTTGGGGAACTCTGC |  |
| Il22 | Fw: CAGCTCCTGTCACATCAGCGGT; | 60 |
|  | Rev: AGGTCCAGTTCCCCAATCGCCT |  |
| Ivl | Fw: AAACTTGGTGAGCCAGAATTACA; | 60 |
|  | Rev: CCTTTCCAGTTGTTTACCCTTCT |  |
| Krt16 | Fw: AGCAGGAGATCGCCACCTA; | 60 |
|  | Rev: AGTGCTGTGAGGAGGAGTGG |  |
| Lce3e | Fw: GCCCTGCTGACTTCTTCTATCCAG; | 63 |
|  | Rev: AGCTACCAGGGAATGAGGACTGTG |  |
| Lcn2 | Fw: CCCTGTATGGAAGAACCAAGGA; | 60 |
|  | Rev: CACACTCACCACCCATTCAGT |  |
| LL37 (Camp) | Fw: CTTCAAGGAACAGGGGGTG; | 60 |
|  | Rev: CCAAGGCAGGCCTACTACTC |  |
| S100a8 | Fw: AAATCACCATGCCCTCTACAAG; | 58 |
|  | Rev: CCCACTTTTATCACCATCGCAA |  |
| S100a9 | Fw: GGTGGAAGCACAGTTGGCA; | 58 |
|  | Rev: GTGTCCAGGTCCTCCATGATG |  |
| Serpinb3a | Fw: TCCTGTTGCCAGTGGAAATCA; | 60 |
|  | Rev: TCAAAGGCATCGACCATTCCC |  |
| Ubc | Fw: AGGTCAAACAGGAAGACAGACGTA; | 60 |
|  | Rev: TCACACCCAAGAACAAGCACA |  |
|  |  |  |

**SUPPLEMENTAL INFORMATION**

## **Methods**

#### Mouse strains and study approval

4-to 9-week-old BALB/c and C57BL/6J mice purchased from Charles River (Sulzfeld, Germany) were kept in the conventional animal facility at the Centre for Medical Research, Medical University of Graz. 6-to 9-week-old nonobese diabetic /severe combined immunodeficient/IL-2 receptor gamma chainnull mice (NSG mice) purchased from The Jackson Laboratory (Maine, USA; strain number 005557) were kept in the specific pathogen free facility at Harvard Medical School. A breeding pair of aryl-hydrocarbon receptor (AhR)-deficient mice (AhR deficient B6.D2N-Ahr^d^/J; strain number 002921 from The Jackson Laboratory) was a generous gift from Dr. Francisco Quintana and mice were bred and housed in the specific pathogen free facility at Harvard Medical School. All experiments were approved by the Austrian Government, Federal Ministry for Science and Research (protocol numbers BMWF-66-010/0032-11/3b-2018 and BMBWF-66.010/0042-V/3b/2019) or the Institutional Animal Care and Use Committee of Brigham and Women’s Hospital (protocol number 2016N000591).

#### Therapeutic agents

For all animal experiments, dithranol in different concentrations (dissolved in vaseline) and vehicle (vaseline cream only) was provided by the pharmacy of the Medical University of Graz, Austria. Recombinant S100 protein (recombinant mouse S100A8/A9 heterodimer, catalogue number 765506) was purchased from Biolegend (San Diego, USA).

#### Dithranol application on BALB/c and AhR-deficient mouse skin and AMP application on C57BL/6J mice

24 h before starting an experiment, dorsal skin of mice was shaved carefully with electric clippers. Dithranol was applied topically on a 2x2 cm area of dorsal skin (40 mg) and right ear (20 mg) of BALB/c mice and concentrations were increased every other day (0.01% on day 1-2, 0.03% on day 3-4 and 0.1% on day 5-6). Control mice were treated similarly with Vaseline cream alone. AhR-deficient mice and C57BL/6 control mice were treated with topical dithranol on dorsal skin and ears and concentrations were increased every other day (0.03% on day 1-2, 0.1% on day 3-4 and 0.3% on day 5-6). Dorsal skin of C57BL/6J mice was topically treated with 5µg of S100a8/a9 protein and double skin fold thickness was measured 24h after application. 24 h after the last topical treatment, all mice were sacrificed and tissues were collected and stored for further analysis.

#### Human-engrafted mouse model

﻿Human skin specimens were grafted onto the backs of NSG mice (The Jackson Laboratory, Maine, USA). Three weeks after engraftment, dithranol dissolved in vaseline (0.1% on day 1-3 and 0.3% on day 4-6) was applied topically on skin grafts. Human skin samples for engraftment were obtained from adult patients undergoing surgical abdominoplasty procedures at the Brigham and Women’s Hospital. The protocols of this study were performed in accordance with the Declaration of Helsinki and were approved by the Institutional Review Board of the Partners Human Research Committee (Partners Research Management). 24 h after the last topical treatment, mice were sacrificed, and skin grafts were collected.

*Monitoring of inflammatory response*

Double skin fold of dorsal skin and ear thickness was measured daily in triplicates before and after topical application of agents using a micrometer (Mitutoyo, Kanagawa, Japan). Erythema index of the skin was assessed using an UV Spectrometer Dermaspect (Cortex Technology, Hadsund, Denmark).

*Transepidermal water loss*

Transepidermal water loss (TEWL) was measured using an open chamber Tewameter® TM 300 probe connected to a Cutometer® dual MPA 580 (Courage and Khazaka Electronic GmbH, Cologne, Germany) by gently applying it on the skin surface for 30s.

#### Analysis of H&E stained sections

Samples were fixed with paraformaldehyde and processed routinely, cut in 4µm sections and stained with hematoxylin and eosin (H&E). Five randomly selected fields per slide were investigated for histological analysis. Thickness of epidermis was measured from basal layer to stratum corneum using an Olympus BX41 microscope (Olympus Life Science Solutions, Hamburg, Germany), cellSens software (Olympus Life Science Solutions) and 20x magnification. Semi-quantitative scoring of infiltrate (0 = none, 0.5 = none/low, 1 = low, 1.5 = low/moderate, 2 = moderate, 2.5 = moderate/high, 3 = high density of infiltrate) was performed at five randomly selected locations per slide at 20x magnification.

#### Gene expression analysis

#### RNA extraction and RT-PCR

Total RNA was extracted from frozen samples using the miRNeasy Mini Kit (Qiagen, Hilden, Germany), according to the manufacturer’s instruction. Per sample, 2µg of RNA was reverse transcribed into cDNA using iScript^TM^ Reverse Transcription Supermix (Bio-Rad, Hercules, CA, USA). Relative gene expression was determined using GoTag® qPCR Master Mix (Promega, Mannheim, Germany) on a CFX384 Touch^TM^ Real-Time PCR Detection System (Bio-Rad). Cycling conditions were Hot-start activation (95°C, 2 min), denaturation for 40 cycles (95°C, 15s) and annealing/extension (60°C, 60s). Samples were run in triplicates and cycle thresholds (CT) were determined and relative mRNA expression to *Ubc* was calculated using the ΔCT method. Primer sequences and annealing temperatures are listed in Supplementary Table 1.

### *Statistical analyses*

As appropriate and indicated in the figure legends, Shapiro-Wilk test, Multiple T-test, unpaired T-test or Kruskal-Wallis test was determined using GraphPad Prism version 8 (GraphPad software, California, USA) in order to test for normality and compare results of treatment groups. Significance was set at a p-value of ≤0.05.
